# Oral-Gut Microbiome Analysis in Patients With Metabolic-Associated Fatty Liver Disease Having Different Tongue Image Feature

**DOI:** 10.3389/fcimb.2022.787143

**Published:** 2022-06-29

**Authors:** Chenxia Lu, Hui Zhu, Dan Zhao, Jia Zhang, Kai Yang, Yi Lv, Miao Peng, Xi Xu, Jingjing Huang, Zuoyu Shao, Mingzhong Xiao, Xiaodong Li

**Affiliations:** ^1^The Clinical Medical College of Traditional Chinese Medicine, Hubei University of Chinese Medicine, Wuhan, China; ^2^Department of Obesity, Hubei Provincial Hospital of Traditional Chinese Medicine, Wuhan, China; ^3^Department of Research and Development, Germountx Company, Beijing, China; ^4^Institute of Liver Disease, Hubei Provincial Academy of Traditional Chinese Medicine, Wuhan, China; ^5^Hubei Key Laboratory of Theoretical and Applied Research of Liver and Kidney in Traditional Chinese Medicine, Hubei Provincial Hospital of Traditional Chinese Medicine, Wuhan, China

**Keywords:** metabolic dysfunction-associated fatty liver disease, tongue coating, oral-intestinal microbiome, 16S rDNA full-length assembly sequencing technology analysis, lab color mode

## Abstract

**Objective:**

The objective of this study was to identify the biological correlation between the tongue coating color and oral and gut micro-characteristics in metabolic-associated fatty liver disease (MAFLD) patients.

**Method:**

The characteristics of the tongue coating were examined using an automatic tongue diagnosis system. Tongue coating and stool samples were collected from 38 MAFLD patients, and 16S rDNA full-length assembly sequencing technology (16S-FAST) was used for bioinformatic analysis.

**Results:**

Twenty-two and 16 subjects were included in two distinct clusters according to the white/yellow color of the tongue coating, which was assessed by the L*a*b* values of the image. Upon analyzing the microorganisms in the tongue coating, 66 and 62 pathognomonic bacterial genera were found in the White and Yellow Coating Groups, respectively. The abundance of *Stomatobaculumis* positively correlated with the a* values of the tongue coating in the White Coating Group, while *Fusobacterium*, *Leptotrichia*, and *Tannerella* abundance was significantly correlated with the b* values in the Yellow Coating Group. Function prediction mainly showed the involvement of protein families related to BRITE hierarchies and metabolism. The MHR (MONO%/high-density lipoprotein cholesterol) of the Yellow Coating Group was higher than that of the White Coating Group.

**Conclusion:**

In MAFLD patients, lower a* values and higher b* values are indicators of a yellow tongue coating. There were also significant differences in the flora of different tongue coatings, with corresponding changes in the intestinal flora, indicating a correlation between carbohydrate metabolism disorders and inflammation in the oral microbiome.

## Introduction

With a global surge in obesity-associated morbidity, non-alcoholic fatty liver disease (NAFLD) has become the most dominant chronic liver disease. NAFLD has a 25% global incidence, resulting in high global health system costs, and approximately 1.7 billion people have been affected (Zhu et al., 2021). In view of the multisystem metabolic dysfunction involving the liver, an international panel of experts has formally renamed NAFLD as metabolic dysfunction-associated fatty liver disease (MAFLD). Although the pathogenesis of this complex disease is not fully elucidated and several major prophylactic and therapeutic barriers remain to be clarified, MAFLD is believed to involve dynamic interactions between genetic susceptibility and environmental factors. As a result of genome-wide association studies, candidate gene and epigenetics research has gradually unveiled factors related to susceptibility, genetic variation, and heterogeneity among patients with MAFLD. Patatin-like phospholipase domain 3 (PNPLA3), transmembrane 6 superfamily member 2, glucokinase regulator isogenetic gene variants, and gene polymorphisms related to inflammation, immunity, metabolism, oxidative stress, adipokines, and muscle kinase have been proven to be significantly associated with MAFLD-related intrahepatic diseases (Kozlitina et al., 2014; Eslam and George, 2016; Eslam et al., 2018). For example, PNPLA3 gene polymorphism is associated with diet pattern, increased sugar intake, and omega-6 polyunsaturated fatty acid intake, and obesity and insulin resistance play a prominent role in MAFLD gene–environment cross talk (Zaharia et al., 2020; Banini et al., 2021).

In traditional Chinese medicine (TCM), the diagnosis of diseases follows the principle of “inspecting exterior to predict interior”, which means the doctor can speculate about changes in diseases inside the body by examining various external signs. The tongue is located in the oral cavity, the first part of the digestive tract, and diseases may affect the tongue coating. The tongue characteristics, which include information about the tongue body, tongue proper, tongue dorsum, and tongue shape, are regarded as a reflection of human health. These characteristics also serve as key evidence for fundamental clinical diagnosis using four diagnostic methods of TCM. However, this information is collected by physicians, and they may be unable to obtain objective or quantitative generalization of the characteristics. Computer image processing technology can be used to segment the images of different parts of the tongue, automatically obtain the spectral parameters of different tongue images, identify the features of the tongue images, and provide objective indicators to describe those features, such as division of area, color, texture, and shape (Zhang et al., 2017). Meanwhile, deep learning can be used to comprehensively interpret tongue images while obtaining a description of the pathological features. The U-net network was used to examine pavement crack images, and the trained model was utilized to assess tongue cracks for further training to avoid the small sample problem (Liu et al., 2012; Xu et al., 2020). Computer-aided tongue diagnosis eliminates the differences caused by environmental and human interference to achieve a standardized result. Although its accuracy depends on continuous improvement by assessing a large number of tongue images, spectral technique and data mining algorithm are applied to explore the physiological and pathological information about the tongue from holistic and systematic perspectives and establish the optical information about tongue image features (Li et al., 2010). The digitized parameters of tongue images have been accepted by various researchers and are gradually being applied in clinical studies; however, the relationship between the tongue image parameters and diseases such as NAFLD, diabetes, and post-cancer diseases has not yet been elucidated. The results of relevant studies are shown in **Table 1** (Zhang et al., 2005; Li et al., 2010; Jiang et al., 2011; Qi et al., 2016; Xue et al., 2018; Woo et al., 2019; Li et al., 2021).

**Table 1:** Recent research about L*a*b* values.

References	Variables	L*a*b* values
Zhang et al., 2005	L*, a*, b* values	L* and a* values were applied to differentiate the light red and red tongue, while L* and b* values were applied for the light red and dark red tongue
Jiang et al., 2011	a*, b* values	The a* and b* values were combined to distinguish the purple and red tongue
Qi et al., 2016	L*, a*, b* values	L* values were used to distinguish the purple and red tongue; a* values were related to the red tongue; b* values were related to different degrees of red tongue.
Xue et al., 2018	L*, a*, b* values	The L* values represented lightness of tongue color; a* and b* values showed shades of red and yellow, respectively.

As a part of the human microbiota, there is a correlation between the oral microbiome and normal oral ecological balance and the occurrence and development of systemic diseases (Gao et al., 2018). The normal oral biofilm is colonized by a complex microbial community in a dynamic equilibrium. Occasionally, the symbiotic relationship is disrupted by lifestyle, immune status, or broad-spectrum antibiotic therapy, and the symbiotic environment of microorganisms is disturbed, resulting in the expansion of opportunistic pathogens and invader species. Such perturbations have been reported to be associated with numerous clinical disorders such as obesity, allergies, and a variety of inflammatory diseases (Costello et al., 2012; Arrieta et al., 2014). Tongue coating microorganisms are important factors affecting the formation of the tongue coating. In recent years, extensive research conducted on the microecology of tongue coating has attracted increasing attention. Studies have shown that the dorsal mucosa of the tongue accumulates abundant bacteria (Rabe et al., 2019), enabling this portion of the tongue to form a relatively complete and independent microecosystem.

The biological mechanism underlying the formation and alteration of the tongue coating is related to the interaction between the microorganisms and the epithelial cells of the dorsal part of the tongue. For example, *Bacillus* was found only in the yellow tongue coating of chronic erosive gastritis patients (Ye et al., 2016). Furthermore, there were significant differences between the dental plaque-derived complexes of *Veillonella* and *Streptococcus* (Kolenbrander et al., 2000). A more clear and thorough understanding of microbial composition on the tongue coating is required for the diagnosis and treatment of different diseases. Wilbert et al. combined species-specific fluorescent tags and high-resolution microscopy to visualize human dorsal tongue microbial communities and highlighted their structure and dynamics (Wilbert et al., 2020). Metagenomic analysis revealed that the variation in abundance of 21 microbial species in the tongue coating microbiota was associated with the occurrence and development of gastritis (Cui et al., 2019). Furthermore, researchers have attempted to study the relationship among different microorganisms in the tongue coating. In this study, standardized tongue images were obtained using specialized equipment. Then, tongue coating and stool samples were collected from 38 MAFLD patients. Cluster analysis showed that the tongue characteristics of MAFLD patients could be clearly divided into two categories by L*a*b* spectral values, which represented white and yellow coating. 16S rDNA full-length assembly sequencing technology analysis revealed the typical variation and differentiation in the tongue-coating microbiome between the two clusters.

## Methods

### Study Subject Recruitment

Thirty-eight MAFLD patients who met the inclusion and exclusion criteria were enrolled in this study from September 2020 to May 2021 at the Hubei Provincial Hospital of TCM (China). Individuals who met the following MAFLD diagnostic criteria were included: MAFLD diagnoses were based on histological (biopsy) and imaging or blood biomarker evidence of fat accumulation in the liver (hepatic steatosis), in addition to one of the following three criteria: overweight/obesity, presence of type 2 diabetes mellitus (T2DM), or evidence of metabolic dysregulation such as the presence of at least two metabolic risk abnormalities: (1) waist circumference (WC) ≥90/80 cm (men/women), (2) blood pressure (BP) ≥130/85 mmHg or specific drug treatment, (3) plasma triglyceride levels (TG) ≥150 mg/dl (≥1.70 mmol/l) or specific drug treatment, (4) plasma high-density lipoprotein (HDL)-cholesterol (HDL-C) levels <40 mg/dl (<1.0 mmol/l) for men and <50 mg/dl (<1.3 mmol/l) for women or specific drug treatment, (5) prediabetes [i.e., fasting glucose levels 100–125 mg/dl (5.6–6.9 mmol/l), or 2-h post-load glucose levels 140–199 mg/dl (7.8–11.0 mmol) or HbA1c content 5.7%–6.4% (39–47 mmol/mol)], (6) homeostasis model assessment of insulin resistance (HOMA-IR) score ≥2.5, and (7) plasma high-sensitivity C-reactive protein (hs-CRP) levels >2 mg/l. The exclusion criteria were as follows: (1) antibiotic, probiotic, or immunosuppressive drug use in the past 2 weeks and (2) the combination of other oral diseases and their complications, such as periodontitis, pulpitis, or oral carcinoma.

### Ethics Statement

This study was approved by the Ethics Committee of Hubei Provincial Hospital of TCM (Ethics No. HBZY2020-C43-01). The protocol used in this study conformed to the Declaration of Helsinki, and all patients and their guardians voluntarily signed the informed consent. Procedures were conducted in compliance with relevant public health laws and privacy regulations.

### Data Collection

This cross-sectional study describes the clinical characteristics of all the enrolled patients. (1) Body weight-related indicators, i.e., body mass index (BMI), body fat percentage, basal metabolic rate, waist-to-hip ratio (WHR), and visceral fat area (VcFA), were assessed using a bioelectric impedance device (InBody 770, InBody, China). These indicators were measured on an empty stomach and without metal attachment. (2) Liver stiffness measurement (LSM) and controlled attenuation parameter (CAP) were determined using a FibroScan^®^ M probe simultaneously to assess liver fibrosis and steatosis *via* evidence-based non-invasive measures (Eddowes et al., 2019). (3) The Homeostasis model assessment-Insulin Resistance (HOMA-IR) score was calculated as fasting plasma glucose (mM) × fasting insulin (mIU/L)/22.5.

### Tongue Image Analysis

The TCM Healthcare Union app (DAOSH Co., Shanghai, China) was used to image the tongue coatings of MAFLD patients under uniform light conditions. Deep learning-based visual tongue qualitative analysis and fractal dimensions were applied to tongue pictures from 38 subjects. In our study, tongue coating color quantitative values were obtained with L*a*b* color mode, developed by the International Commission on Lighting (CIE), in which color space is larger than that in the RGB and CMYK mode. The L*a*b* color model consists of three elements; *L* shows brightness, while *a* and *b* show gradation of color. A higher *a* value indicates a redder tongue color; a higher *b* value indicates a yellower tongue color.

### Sample Collection

Blood samples were collected on an empty stomach and at multiple time points to determine the clinical, biochemical, and metabolic signatures of MAFLD individuals at the initial visit, using the oral glucose tolerance test (OGTT), insulin provocation, hs-CRP level, liver function test, and blood lipid level.

Subjects were instructed not to eat or drink colored foods or beverages (except water) during the 8 h before sampling. Brushing of tongues was not allowed 2 h before sampling. The tongue dorsa of subjects were sampled by gently scraping the dorsal surface of the tongue from back to front using a swab and then putting the swab into a collection tube containing a DNA preservation solution (Swab DNA Storage Tube, CwBiotech, Beijing, China). Fecal DNA storage tubes (CW2654, CwBiotech, Beijing, China) were provided to patients for stool collection in the morning after ≥8 h of fasting. Tongue dorsum and fecal samples were finally stored at -25°C for further processing.

### DNA Extraction and 16S Full-Length Library Construction

Bacterial DNA from the tongue dorsum and fecal samples was extracted using a swab genomic DNA extraction kit (CW2654, CwBiotech, Beijing, China) and an intestinal DNA extraction kit, respectively (TIANamp Stool DNA Kit, DP328, Tiangen Biotech, Beijing, China). The 16S rDNA full-length assembly sequencing technology (16S FAST) was used to perform further classifications to reach a species level (Karst et al., 2018) by assaying DNA sequences encoding bacterial ribosomal 16S RNA, including 9 variable regions and 10 conserved regions. Qualitative and quantitative analyses as well as quality control were performed using 10 ng DNA. The splice and link libraries were then built. Next, data from electrophoresis and the measurements of Qubit concentrations were assembled for quality control before Illumina NovaSeq 6000 sequencing (Illumina, USA). The detailed process was reported in a previously published study (Dong et al., 2021).

### Bioinformatic Analysis

The Unique Molecular Identifier (UMI) pairing relationship was extracted *via* the connecting library. All sequences corresponding to each paired UMI were obtained from the splicing library. Cutadapt V1.2.1 was applied to remove primers and UMI tags for each paired UMI sequence, and SPAdes V3.13.1 software was used for assembly with default parameters to obtain the full-length 16S sequence for each UMI. The reads were classified with a taxonomy-finding algorithm assigned to operational taxonomic units (OTUs) within the software package Mothur V1.42.0 to determine the microbial communities of individuals. Alpha diversity at the genus level was measured with Simpson and Shannon indices derived using the QIIME1.8.0 tool. The beta diversity was measured at the genus level with principal component analysis (PCoA) using R3.6.1 package vegan2.5-3. The OTU table was compared with the (Kyoto Encyclopedia of Genes and Genomes) KEGG database to obtain metabolic pathway information for each OTU. The functional potential of the tongue microbiome was predicted by Statistical Analysis of Metagenomic Profiles (PICRUSt), and it was used to construct the gene function prediction spectra of the whole lineages of archaeal and bacterial domains.

### Statistical Analysis

Data analysis was performed with SPSS v. 25.0 software (IBM Corp., Armonk, NY, USA). The normality of the variable distribution and homogeneity of variances were determined with the Shapiro–Wilk and Levene tests, respectively.

In the case of normal distribution and homogeneity of variance, a t-test was used. Otherwise, a non-parametric test was performed. The comparison of categorical variables by Fisher’s exact test and Wilcoxon rank-sum test was applied to categorical variables and continuous variables. The associations between independent variables were analyzed *via* Spearman’s rank test, and the *p-*values were corrected *via* the *Bonferroni* correction for multiple comparisons.

## Results

### Characteristics of the Patients

**Figure 1:**
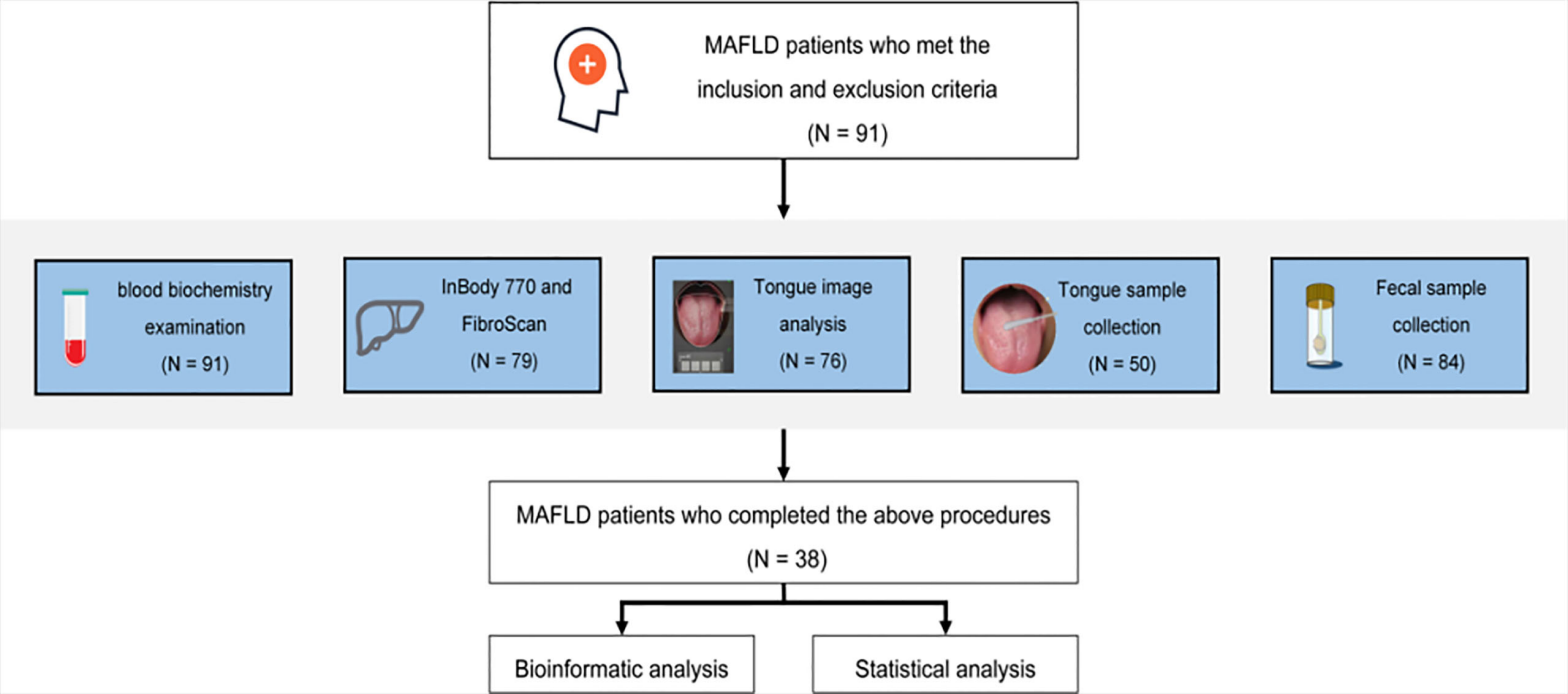
Program flowchart.

The basic, biochemical, and microbiome composition-related information of 91 patients was collected. Ultimately, 38 patients who completed tongue imaging and information recognition analysis were included in the follow-up study (**Figure 1**). Further research statistics showed that most patients with MAFLD were middle-aged males.

**Figure 2:**
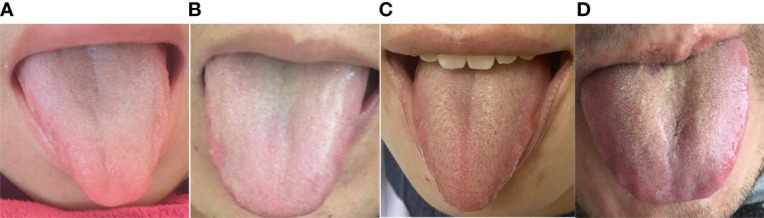
Images of the two groups. **(A)** Female with an a* value of 7.46 and b* value of 3.65. **(B)** Male with an a* value of 8.46 and b* value of 3.71. Both **(A)** and **(B)** are in the White Coating Group. **(C)** Female with an a* value of 15.82 and b* value of 10.72. **(D)** Male with an a* value of 13.83 and b* value of 13.28. Both **(C, D)** are in the Yellow Coating Group.

The L*a*b* parameters of coating color for the overall tongue imaging of the patients were compared, and these patients were divided into two groups by system cluster analysis with *SPSS* (**Table 2**). There were significant differences between the two clusters (*p = 0.000)*; the cluster with higher a* and b* values tended to exhibit a yellow tongue coating, while those with higher a* values tended to have a red tongue color. The other cluster with lower values could be regarded as a white coating, and representative pictures of the two clusters are shown in **Figure 2**.

**Table 2:** Characteristics of the two groups.

Group	L*a*b* color model parameters of tongue coating		
	L*	a*	b*
White Coating Group	66.73 ± 7.76	9.15 ± 3.32	1.80 ± 3.45
Yellow Coating Group	48.32 ± 9.52	16.88 ± 2.94	9.53 ± 3.81
*p-*value	0.000	0.000	0.000

In the two groups, some individuals had abnormal biochemical indices and increased levels of metabolic disease risk factors. Compared to the Yellow Coating Group, the White Coating Group showed higher abnormal levels of ALT and less insulin resistance. However, there were no significant differences (*p* > 0.05, **Table 3**), except that the MONO% and MHR (MONO%/HDL-C) in the Yellow Coating Group were higher than those of the White Coating Group. Furthermore, all patients in the Yellow Coating Group were obese (BMI ≥ 28) (Dewulf et al., 2013).

**Table 3:** Characteristics of MAFLD patients in the two groups.

Characteristic	White Coating Group (n = 22)	Yellow Coating Group (n = 16)	*p*
Age in years, median (min–max)	30 (22–34)	30 (21–38)	0.756
Gender			
Male n (%)	16 (72.73)	8 (50)	0.187
Female n (%)	6 (27.27)	8 (50)	
Severity of fatty liver			
Mild fatty liver, n (%)	7 (31.82)	6 (37.50)	0.120
Moderate fatty liver, n (%)	10 (45.45)	10 (62.50)	
Severe fatty liver, n (%)	5 (22.73)	0 (0.00)	
ALT, U/L, median (min–max)	46.5 (20.75, 71)	33.50 (24.00, 95.25)	0.976
AST, U/L, median (min–max)	28 (20.75, 39.25)	26.00 (20.00, 41.25)	0.745
CHOL, mmol/L, median (min–max)	4.88 (4.16, 5.66)	4.51 (4.08, 5.36)	0.383
TG, mmol/L, median (min–max)	1.69 (1.10, 2.32)	1.69 (1.27, 2.29)	0.894
HDL-C, mmol/L, median (min–max)	1.06 (0.95, 1.15)	1.00 (0.99, 1.21)	0.756
LDL-C, mmol/L, median (min–max)	3.12 (2.48, 3.94)	2.66 (2.33, 3.36)	0.132
HOMA-IR, mmol/L, median (min–max)	3.83 (2.38, 7.14)	4.69 (3.34, 7.61)	0.533
MONO%, median (min–max)	5.5 (4.93, 6.55)	7.15 (6.43, 8.38)	0.010
MHR (MONO%/HDL-C)	5.01 (4.27, 6.36)	6.34 (5.60, 9.55)	0.019
Weight (kg)	97.46 ± 19.89	92.50 ± 12.17	0.383
Protein (kg)	12.02 ± 1.95	11.17 ± 2.05	0.206
Minerals (kg)	4.15 ± 0.64	3.89 ± 0.66	0.235
BFM (kg)	36.78 ± 13.54	35.89 ± 6.79	0.810
SLM (kg)	57.24 ± 9.42	53.37 ± 9.56	0.222
FFM (kg)	60.67 ± 9.93	56.60 ± 10.10	0.224
SMM (kg)	34.29 ± 5.91	31.75 ± 6.22	0.209
BMI (kg/m^2^)	32.93 ± 5.66	32.65 ± 3.09	0.856
Overweight n (%)	4 (18.18)	0 (0)	0.124
Obesity n (%)	18 (81.82)	16 (100)	
PBF (%)	36.94 ± 8.07	38.90 ± 6.06	0.418
BMR (kJ/m^2^·h)	1681 ± 214	1593 ± 218	0.224
WHR	0.98 ± 0.08	0.97 ± 0.05	0.566
VFA (cm^2^)	165.09 ± 61.69	166.05 ± 35.27	0.956
Circumference of the neck (cm)	40.40 ± 3.38	39.80 ± 2.85	0.563

AST, aspartate aminotransferase; ALT, alanine aminotransferase; CHOL, cholesterol; TG, triglyceride; HDL-C, high-density lipoprotein cholesterol; LDL-C, low-density lipoprotein cholesterol; MONO, monocytes; HOMA-IR, homeostasis model assessment-insulin resistance; BFM, body fat mass; SLM, soft lean mass; FFM, fat-free mass; SMM, skeletal muscle mass.

### Patient Microbiome Information

**Figure 3:**
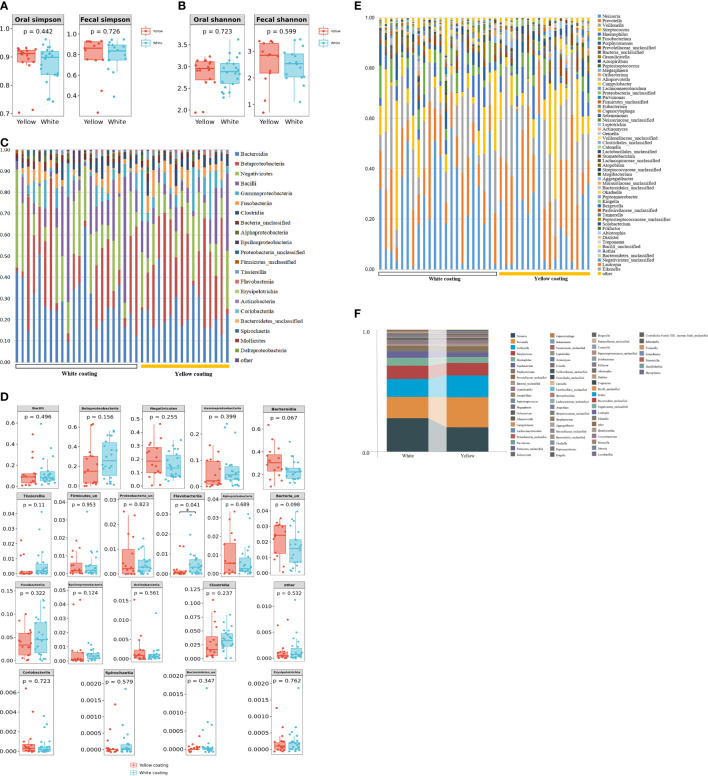
Tongue coating and intestinal microbial information from MAFLD patients. Microbial diversity, which was calculated by the Shannon index **(A)** and Simpson index **(B)**, showed no significant differences between the two groups (*p* > 0.05). **(C, D)** showed a statistically different flora at the class level. **(E, F)** showed a statistically different flora at the genus level.

16S DNA full-length sequencing was conducted on fecal and oral samples from 38 MAFLD patients to characterize the microbial community diversity. A total of 18,407 contigs were obtained in oral samples, while 18,351 contigs were obtained in fecal samples. There was no statistical significance in terms of Shannon and Simpson indices of genus between the two different groups in amplicon sequence variant (ASV) (*p > 0.05*, **Figures 3A, B**). In terms of *class* level, there were 22 classes in all samples; however, Mollicutes and Deltaproteobacteria only existed in the White Coating Group. Meanwhile, Flavobacteriia was statistically more abundant in the Yellow Coating Group than in the White Coating Group (*p = 0.041*, **Figures 3C, D**).

In terms of genus level, upon the taxonomic analysis of oral microbiomes, 66 and 62 bacterial genera were found in the White Coating Group and Yellow Coating Group, respectively. *Corynebacterium*, *Moraxella*, *Ottowia*, *Lactobacillus*, *Johnsonella*, *Tissierella*, and *Enterobacter* were only found in the White Coating Group, while *Shuttleworthia*, *Simonsiella*, *Desulfobulbus*, and *Mycoplasma* were unique to the Yellow Coating Group (**Figure 3D**). In terms of relative abundance, *Neisseria* was dominant in the White Coating Group, while *Prevotella* was dominant in the Yellow Coating Group (**Figures 3E, F**).

### Correlation Between Microbiome and Tongue Parameters

**Figure 4:**
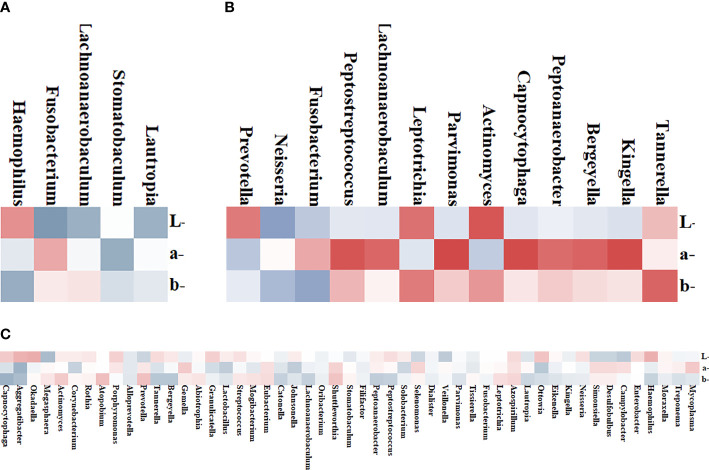
Heat map of the correlation between tongue coating bacteria and the tongue image parameters. Red represents positive correlation and blue represents negative correlation; the darker the color, the stronger the correlation. **(A)** Four genera in the White Coating Group that were significantly related to L*a*b* values. **(B)** Thirteen genera in the Yellow Coating Group significantly related to L*a*b* values. **(C)** Tongue flora in both groups significantly related to L*a*b* values.

To further explore the correlation between tongue image parameters and tongue coating colonies, we conducted a correlation analysis. Among the 51 shared genera of tongue coating bacteria in 38 patients, *Capnocytophaga* (b* values, *R = 0.440*, **Figure 4C**), *Aggregatibacter* (a*/b* values, *R = 0.434/0.374*, **Figure 4C**), *Okadaella* (L*values, *R = 0.327*, **Figure 4C**), and *Megasphaera* (L*values, *R = 0.378*, **Figure 4C**) were significantly correlated with tongue image parameters.

In the White Coating Group, *Haemophilus*, *Fusobacterium*, *Lachnoanaerobaculum*, *Stomatobaculum*, and *Lautropia* and tongue coating color parameters were significantly correlated. Among them, *Stomatobaculum* (*R* = 0.461, **Figure 4A**) was correlated with a* values of the whole tongue coating. In the Yellow Coating Group, *Fusobacterium* (*R = 0.526*, **Figure 4B**), *Leptotrichia* (*R = 0.521*, **Figure 4B**), *Tannerella* (*R = 0.611*, **Figure 4B**), and the b*values of tongue coating color parameters were significantly correlated.

### Characteristics of Intestinal Microflora in Patients

**Figure 5:**
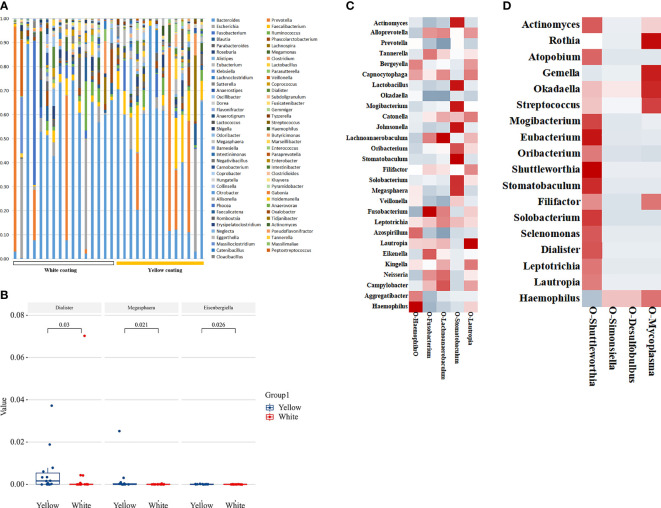
Distribution of intestinal flora. **(A)** The distribution of different groups at the genus level is shown in the bar chart. **(B)** Bacterial communities with statistical differences between the two groups are shown in the boxplot. In **(C, D)**, the correlation between tongue coating bacteria and intestinal bacteria in the White Coating Group and Yellow Coating Group, respectively, are shown in the heatmap.

To estimate the structure of the gut microbiome, we studied 29 individual 16S DNA full-length sequencing results of microbial DNA obtained from all 38 patients. In total, 137 genera were identified in this cohort. Among them, 89 common genera existed in two groups, and the others were only found in one of two groups (**Figure 5A**). Of all these genera, the relative abundance of *Dialister*, *Megasphaera*, and *Eisenbergiella* showed significant differences between the two groups (*p = 0.03*, *p = 0.021*, *p = 0.026*, **Figure 5B**), while *Eisenbergiella* was only found in the Yellow Coating Group. Those intestinal flora, as shown in the heat map, were closely related to the tongue coating flora, proving its impact on tongue coating parameters in the respective group. According to correlation statistical analysis, oral *Stomatobaculum* was highly associated with the species of intestinal flora in the White Coating Group (**Figure 5C**). Meanwhile, oral *Shuttleworthia* and *Mycoplasma* were highly correlated with intestinal flora in the Yellow Coating Group (**Figure 5D**).

### PICRUSt Function Predictive Analysis

**Figure 6:**
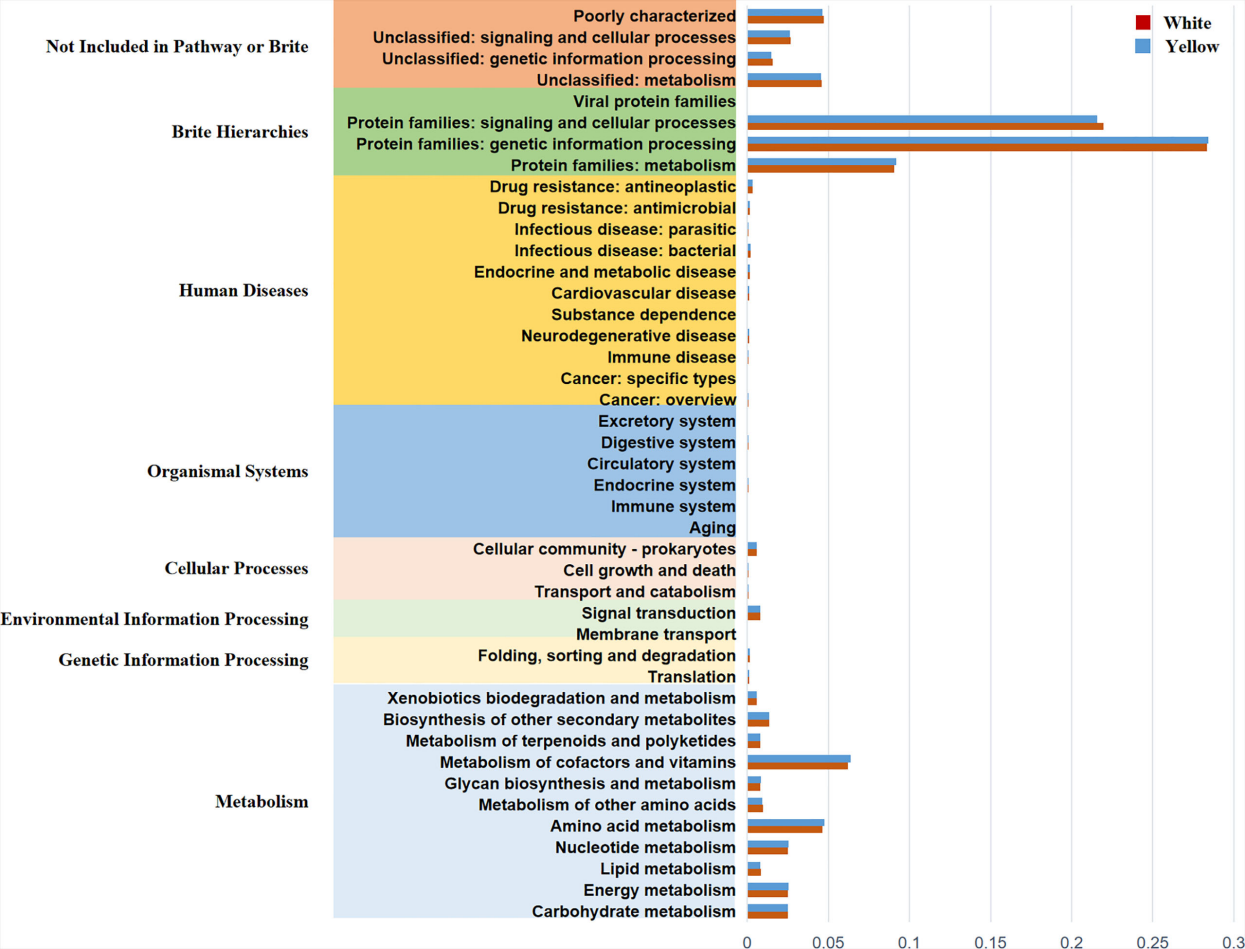
Histogram of functional prediction for different groups of bacteria.

In order to predict and compare the functions of the White Coating Group and Yellow Coating Group, PICRUS software was used to predict the functions based on the KEGG database with an OTU table of tongue coating bacteria. Function prediction mainly involves protein families, genetic information processing, signaling, and cellular processes. The results showed that there were 11 pathways related to metabolism, which were mainly related to the metabolism of cofactors and vitamins, amino acids, and energy (**Figure 6**).

## Discussion

Tongue disease diagnosis is an important aspect of TCM. However, traditional tongue disease diagnosis methods, including direct observation of the tongue, have limitations because of various external and subjective factors (Kim et al., 2014). The in-depth application of computer and image processing technology has facilitated a more objective analysis of the MAFLD patient tongue image, which is helpful in disease determination using TCM.

The L*a*b* color model was developed by CIE (International Lighting Committee). The L*a*b* color model utilizes a coordinate system (Kainuma et al., 2015). Upon comparing the characteristics of moss color in different color modes of L*a*b*, the a*and b* values for the 22 patients in the Yellow Coating Group were significantly larger than those in the White Coating Group; tongue coating color was more associated with yellow, indicating obvious fever. In addition, the BMI of Yellow Coating Group patients met the diagnostic criteria for obesity. Combined with the b* values, it can be inferred that the thermal image in obese people is heavier than that in non-obese individuals.

Endocrine system diseases are closely associated with tongue coating. Further analysis showed that the oral microbiota of patients in this study showed two major “stomatotypes” in the oral microbiome, which included *Neisseria* and *Prevotella* as the main bacteria (Willis et al., 2018). Studies have shown that interleukin-6 (IL-6) expression is increased in human dental pulp cell cultures stimulated with *Prevotella* intermedia lipopolysaccharide, while the changes in IL-6 and HDL-C were related with carbohydrate diet (Sarkar et al., 2021). *Actinomycetes* and *Prevotella* may serve as key strains to distinguish between physiological and pathological yellow greasy coatings. Alterations in the tongue coating resulting from disease reflect changes in the tongue microbes. Li found the correlation between the changes in oral microbial flora and the formation mechanism of a greasy coating using denaturing gradient gel electrophoresis technology to detect the microbial flora of the tongue coating in the greasy group, non-greasy group, and normal group of chronic gastritis patients (Li et al., 2012). Our study also showed that *Shuttleworthia*, *Simonsiella*, *Desulfobulbus*, and *Mycoplasma* only appeared in MAFLD patients with yellow moss. These genera are found in patients with dental caries, resulting from biofilm-induced acidification in response to dietary carbohydrates (Wang et al., 2019). In the study of the relationship of gut microbial carbohydrate metabolism with weight loss, increased abundance of *Dialister* in patients who lost 5% of their body weight was found (Muñiz Pedrogo, 2018). This was related to our finding that Yellow Coating Group patients were all obese with more *Dialister* than other groups. Tongue microorganisms are one of the agents that lead to the formation of different coatings.

Upon exploring oral microflora and L*a*b* parameters, our analysis of the correlation between intestinal and tongue flora in different groups found a strong correlation between the a* values and *Stomatobaculum. tomatobaculum* is able to convert glucose into butyrate, lactate, isovalerate, and acetate as major metabolic end products (Sizova et al., 2013). High a* values were a characteristic of the Yellow Coating Group, which means that the patients in this group tend to have a red tongue covered with yellow moss. This also means that the patients in the Yellow Coating Group have a deeper heat constitution than those of the White Coating Group. *Leptotrichia* in the Yellow Coating Group was able to ferment carbohydrates, producing lactic acid as its major metabolic end product.

The relationship between tongue coating colonies and intestinal flora in the same host has been demonstrated widely. *Shuttleworthia* in the tongue coating and the intestinal flora was also highly positively correlated in the Yellow Coating Group. *Shuttleworthia* also plays an important role in the metabolism of glucose to short-chain fatty acids and presents the advantages of duodenal ecological imbalance and conversion to specific potential pathogenic bacteria (Luca Maccioni et al., 2021). This also proves the relationship between liver flora and liver metabolism. *Simonsiella* plays the same role in carbohydrate metabolism, and it has been demonstrated that the dietary intake of *Simonsiella* carriers is generally excessive (Gregory et al., 1985). *Shuttleworthia* and *Simonsiella*, which have the same metabolic effect, had the same related flora in the intestine, but their correlation results were quite opposite. Their symbiotic relationship in the oral cavity needs to be further explored in our follow-up study. In the meantime, *Mycoplasma* in the tongue coating uniformly correlates with *Rothia*, *Atopobium*, *Gemella*, *Okadaella*, and *Streptococcus* in the gut. *Mycoplasma*, which are clinically important components of the human pathogenic microbiome in various tissues, are related with *Atopobium* that coexist in intramucosal tumors and significantly increase in multiple polyp adenomas. Another study showed that the risk of colorectal cancer was increased in patients with bacteremia from *Gemella morbillorum. Mycoplasma* may be a significant predictive factor for gastrointestinal cancer. There are also significant differences in the flora and characteristic functions of tongue coating in patients with yellow fur, which is of guiding significance for the dialectical medication of TCM based on network pharmacology.

Monocytes are immune cells derived from bone marrow precursors, which play an important role in immune monitoring and inflammatory response. Under the stimulation of lipopolysaccharide, typical monocytes secrete a large amount of granulocyte colony-stimulating factor and IL-6 (Wong et al., 2011). LPS, as the active component of gram-negative membrane and endotoxin, can be produced in large quantities by bacteria (Gao et al., 2021). Combined with higher MONO% results in the Yellow Coating Group, the red tongue covered with yellow moss might be the manifestation of excessive intake of carbohydrates, making the oral environment more conducive to the growth of lipopolysaccharide bacteria, aggravating inflammation in the body, and showing the heat symptoms estimated by TCM. HDL-C had been found to reverse the transport of cholesterol out of cells. Increasing HDL-C could reduce inflammation and insulin resistance, and reducing the MHR level could help to reduce insulin resistance (Han et al., 2021). The same high MHR and similar trend of HOMA-IR were observed in the Yellow Coating Group, which consisted of all obese patients. In obese patients, inflammatory factors increase and aggravate insulin resistance (Gastaldelli et al., 2017). This result could be further verified in subsequent studies with larger sample size.

In general, compared to the tongues of patients with smaller *a*-mean and *b*-mean values, the tongues of MAFLD patients with higher *b*-mean values had a deeper yellow color. There were significant differences between the two characteristic microbiomes, and the enrichment of bacteria related to carbohydrate metabolism could be seen in patients with yellowish tongue.

Our study has limitations as a result of its retrospective cross-sectional design without metabolomics analysis of microorganisms to explain the different internal mechanisms of the genera. Currently, tongue images and flora samples from patients are being continuously collected. At the same time, the serum and urine of each patient have also been preserved for further metabolomic analysis and verification of the above research results. However, most patients with MAFLD have no obvious symptoms. In the field of TCM, further guidelines related to tongue image information will be needed in the future. Overall, our study preliminarily confirmed that color formation in the tongue coating is related to microbial metabolism; these findings can provide a theoretical basis for the objective analysis of tongue color.

## Data Availability Statement

The original contributions presented in the study are publicly available. These data can be found here: PRJNA770736.

## Ethics Statement

The studies involving human participants were reviewed and approved by the Ethics Committee of Hubei Hospital of Traditional Chinese Medicine. The patients/participants provided their written informed consent to participate in this study.

## Author Contributions

XL and MX designed the study and revised the manuscript. CL and HZ contributed significantly to data analyses and wrote the manuscript. CL, HZ, DZ, JZ, YL, XX, JH, and MP contributed to clinical data collection and structured logging. KY performed the oral-gut microbiome sequencing. ZS collected the biological samples. All authors contributed to the article and approved the submitted version.

## Funding

This research was supported by a grant from the Key Project Natural Science Foundation of Hubei Province (No. 2020CFA023).

## Conflict of Interest

Author KY was employed by Germountx Company. The remaining authors declare that the research was conducted in the absence ofany commercial or financial relationships that could be construed as a potentialconflict of interest.

## Publisher’s Note

All claims expressed in this article are solely those of the authors and do not necessarily represent those of their affiliated organizations, or those of the publisher, the editors and the reviewers. Any product that may be evaluated in this article, or claim that may be made by its manufacturer, is not guaranteed or endorsed by the publisher.

## References

[B1] Arrieta M. C. Stiemsma L. T. Amenyogbe N. Brown E. M. Finlay B. (2014). The Intestinal Microbiome in Early Life: Health and Disease. Front. Immunol. 5. doi: 10.3389/fimmu.2014.00427 PMC4155789 25250028

[B2] Banini B. A. Kumar D. P. Cazanave S. Seneshaw M. Mirshahi F. Santhekadur P. K.. (2021). Identification of a Metabolic, Transcriptomic, and Molecular Signature of Patatin-Like Phospholipase Domain Containing 3-Mediated Acceleration of Steatohepatitis. Hepatology 73 (4), 1290–1306. doi: 10.1002/hep.31609 33131062 PMC8046714

[B3] Costello E. K. Stagaman K. Dethlefsen L. Bohannan B. J. M. Relman D. A. (2012). The Application of Ecological Theory Toward an Understanding of the Human Microbiome. Science. 336, 1255–1262. doi: 10.1126/science.1224203 22674335 PMC4208626

[B4] Cui J. Cui H. Yang M. Du S. Li J. Li Y.. (2019). Tongue Coating Microbiome as a Potential Biomarker for Gastritis Including Precancerous Cascade. Protein Cell 10, 496–509. doi: 10.1007/s13238-018-0596-6 30478535 PMC6588651

[B5] Dewulf E. M. Cani P. D. Claus S. P. Fuentes S. Puylaert P. G. B. Neyrinck A. M.. (2013). Insight Into the Prebiotic Concept: Lessons From an Exploratory, Double Blind Intervention Study With Inulin-Type Fructans in Obese Women. Gut 62, 1112–1121. doi: 10.1136/gutjnl-2012-303304 23135760 PMC3711491

[B6] Dong S. Jiao J. Jia S. Li G. Zhang W. Yang K.. (2021). 16s rDNA Full-Length Assembly Sequencing Technology Analysis of Intestinal Microbiome in Polycystic Ovary Syndrome. Front. Cell. Infect. Microbiol. 11. doi: 10.3389/fcimb.2021.634981 PMC8141595 34041041

[B7] Eddowes P. J. Sasso M. Allison M. Tsochatzis E. Anstee Q. M. Sheridan D.. (2019). Accuracy of FibroScan Controlled Attenuation Parameter and Liver Stiffness Measurement in Assessing Steatosis and Fibrosis in Patients With Nonalcoholic Fatty Liver Disease. Gastroenterology 156, 1717–1730. doi: 10.1053/j.gastro.2019.01.042 30689971

[B8] Eslam M. George J. (2016). Genetic and Epigenetic Mechanisms of NASH. Hepatol. Int. 10, 394– 406. doi: 10.1007/s12072-015-9689-y 26683320

[B9] Eslam M. Valenti L. Romeo S. (2018). Genetics and Epigenetics of NAFLD and NASH: Clinical Impact. J. Hepatol. 68, 268–279. doi: 10.1016/j.jhep.2017.09.003 29122391

[B10] Gao J. Guo X. Wei W. Li R. Hu K. Liu X.. (2021). The Association of Fried Meat Consumption With the Gut Microbiota and Fecal Metabolites and Its Impact on Glucose Homoeostasis, Intestinal Endotoxin Levels, and Systemic Inflammation: A Randomized Controlled-Feeding Trial. Diabetes Care 44 (9), 1970–1979. doi: 10.2337/dc21-0099 34253560

[B11] Gao L. Xu T. Huang G. Jiang S. Gu Y. Chen F. (2018). Oral Microbiomes: More and More Importance in Oral Cavity and Whole Body. Protein Cell 9, 488–500. doi: 10.1007/s13238-018-0548-1 29736705 PMC5960472

[B12] Gastaldelli A. Gaggini M. DeFronzo R. A. (2017). Role of Adipose Tissue Insulin Resistance in the Natural History of Type 2 Diabetes: Results From the San Antonio Metabolism Study. Diabetes 66 (4), 815–822. doi: 10.2337/db16-1167 28052966

[B13] Gregory D. A. Kuhn D. A. Daly K. R. Flygenring K. (1985). Statistical Association of Dietary Components With Simonsiella Species Residing in Normal Human Mouths. Appl. Environ. Microbiol. 50, 704–705. doi: 10.1016/0141-4607(85)90029-0 4073897 PMC238695

[B14] Han X. Ying C. J. Zhou X. Y. Ling H. W.. (2021). Relationship Between Monocyte/High-Density Lipoprotein Cholesterol Ratio and Insulin Resistance in Patients With Type 2 Diabetes Mellitus. Chin. J. Diabetes 29 (09), 659–664.

[B15] Jiang Y. H. Jiang Z. Y. Xu B. Ma J. Zhang J. Y. Hu P.. (2011). Analysis of the Characteristics of Tongue Image in 76 Children With Mycoplasma Pneumonia [J]. Liaoning J. Traditional Chin. Med. 38 (10), 3.

[B16] Kainuma M. Furusyo N. Urita Y. Nagata M. Ihara T. Oji T.. (2015). The Association Between Objective Tongue Color and Endoscopic Findings: Results From the Kyushu and Okinawa Population Study (KOPS). BMC Complement. Altern. Med. 15, 372. doi: 10.1186/s12906-015-0904-0 26474972 PMC4609076

[B17] Karst S. M. Dueholm M. S. McIlroy S. J. Kirkegaard R. H. Nielsen P. H. Albertsen M. (2018). Retrieval of a Million High-Quality, Full-Length Microbial 16S and 18S rRNA Gene Sequences Without Primer Bias. Nat. Biotechnol. 36, 190–195. doi: 10.1038/nbt.4045 29291348

[B18] Kim J. Han G. Ko S. J. Nam D. H. Park J. W. Ryu B.. (2014). Tongue Diagnosis System for Quantitative Assessment of Tongue Coating in Patients With Functional Dyspepsia: A Clinical Trial. J. Ethnopharmacol. 155, 709–713. doi: 10.1016/j.jep.2014.06.010 24933221

[B19] Kolenbrander P. E. Palmer R. J. Rickard A. H. Jr. Jakubovics N. S. Chalmers N. I. Diaz P. I.. (2000). Bacterial Interactions and Successions During Plaque Development. Periodontology 42, 47–79. doi: 10.1111/j.1600-0757.2006.00187.x 16930306

[B20] Kozlitina J. Smagris E. Stender S. Nordestgaard B. G. Zhou H. H. Tybjærg-Hansen A.. (2014). Exome-Wide Association Study Identifies a TM6SF2 Variant That Confers Susceptibility to Nonalcoholic Fatty Liver Disease. Nat. Genet. 46, 352–356. doi: 10.1038/ng.2901 24531328 PMC3969786

[B21] Liu Z. Wang H. Li Q. (2012). Tongue Tumor Detection in Medical Hyperspectral Images. Sensors 12. doi: 10.3390/s120100162 PMC3279206 22368462

[B22] Li Q. Wang Y. Liu H. Sun Z. Zhi L. (2010). Tongue Fissure Extraction and Classification Using Hyperspectral Imaging Technology. Appl. Opt. 49, 2006–2013. doi: 10.1364/AO.49.002006 20389998

[B23] Li J. Yuan P. Hu X. Huang J. Cui L. Cui J.. (2021). A Tongue Features Fusion Approach to Predicting Prediabetes and Diabetes With Machine Learning. J. Biomed. Inform. 115, 103693. doi: 10.1016/j.jbi.2021.103693 33540076

[B24] Li F. Zhao J. Pang X. (2012). The Oral Microbial Fingerprint on the Greasy Tongue Coating of Patients With Chronic Gastritis. Zhongguo Zhong Xi Yi Jie He Za Zhi Zhongguo Zhongxiyi Jiehe Zazhi Chin. J. Integr. Tradit. West. Med. 32, 1331–1335. 23163140

[B25] Mar Tomás M. Bou G. (2011) Molecular Detection of Human Bacterial Pathogens. Available at: http://www.mendeley.com/research/molecular-detection-human-bacterial-pathogens/ (Accessed September 15, 2021). CRC Press.

[B26] Qi Z. Tu L. P. Chen J. B. Hu X. J. Xu J. T. Zhang Z. F.. (2016). The Classification of Tongue Colors With Standardized Acquisition and ICC Profile Correction in Traditional Chinese Medicine. BioMed. Res. Int. 2016, 3510807. doi: 10.1155/2016/3510807 28050555 PMC5168476

[B27] Rabe A. Gesell Salazar M. Michalik S. Fuchs S. Welk A. Kocher T.. (2019). Metaproteomics Analysis of Microbial Diversity of Human Saliva and Tongue Dorsum in Young Healthy Individuals. J. Oral. Microbiol. 11, 1654786. doi: 10.1080/20002297.2019.1654786 31497257 PMC6720020

[B28] Sarkar A. Kuehl M. N. Alman A. C. Burkhardt B. R. (2021). Linking the Oral Microbiome and Salivary Cytokine Abundance to Circadian Oscillations. Sci. Rep. 11 (1), 2658. doi: 10.1038/s41598-021-81420-3 33514800 PMC7846843

[B29] Sizova M. V. Muller P. Panikov N. Mandalakis M. Hohmann T. Hazen A.. (2013). Stomatobaculum Longum Gen. Nov., Sp. Nov., an Obligately Anaerobic Bacterium From the Human Oral Cavity. Int. J. Syst. Evol. Microbiol. 63, 1450–1456. doi: 10.1099/ijs.0.042812-0 22843721 PMC3709536

[B30] Wang Y. Wang S. Wu C. Chen X. Duan Z. Xu Q.. (2019). Oral Microbiome Alterations Associated With Early Childhood Caries Highlight the Importance of Carbohydrate Metabolic Activities. mSystems 4, e00450–e00419. doi: 10.1128/mSystems.00450-19 31690590 PMC6832018

[B31] Wilbert S. A. Mark Welch J. L. Borisy G. G. (2020). Spatial Ecology of the Human Tongue Dorsum Microbiome. Cell Rep. 30, 4003–4015.e3. doi: 10.1016/j.celrep.2020.02.097 32209464 PMC7179516

[B32] Willis J. R. González-Torres P. Pittis A. A. Bejarano L. A. Cozzuto L. Andreu-Somavilla N.. (2018). Citizen Science Charts Two Major “Stomatotypes” in the Oral Microbiome of Adolescents and Reveals Links With Habits and Drinking Water Composition. Microbiome 6, 218. doi: 10.1186/s40168-018-0592-3 30522523 PMC6284318

[B33] Wong K. L. Tai J. J. Wong W. C. Han H. Sem X. Yeap W. H.. (2011). Gene Expression Profiling Reveals the Defining Features of the Classical, Intermediate, and Nonclassical Human Monocyte Subsets. Blood 118 (5), e16–e31. doi: 10.1182/blood-2010-12-326355 21653326

[B34] Woo J. Xing F. Prince J. L. Stone M. Green J. R. Goldsmith T.. (2019). Differentiating Post-Cancer From Healthy Tongue Muscle Coordination Patterns During Speech Using Deep Learning. J. Acoust. Soc Am. 145, EL423. doi: 10.1121/1.5103191 31153323 PMC6530633

[B35] Xue Y. Duan J. Wang X. Ding D. Wu Y. Gu S.. (2018). Shanghai J. Traditional Chin. Med. 52 (6), 4.

[B36] Xu Q. Zeng Y. Tang W. Peng W. Xia T. Li Z.. (2020). Multi-Task Joint Learning Model for Segmenting and Classifying Tongue Images Using a Deep Neural Network. IEEE J. Biomed. Health Inform. 24, 2481–2489. doi: 10.1109/JBHI.2020.2986376 32310809

[B37] Ye J. Cai X. Yang J. Sun X. Hu C. Xia J.. (2016). Bacillus as a Potential Diagnostic Marker for Yellow Tongue Coating. Sci. Rep. 6, 32496. doi: 10.1038/srep32496 27578261 PMC5006162

[B38] Zaharia O. P. Strassburger K. Knebel B. Kupriyanova Y.. (2020). Role of Patatin-Like Phospholipase Domain-Containing 3 Gene for Hepatic Lipid Content and Insulin Resistance in Diabetes. Diabetes Care 43 (9), 2161–2168. doi: 10.2337/dc20-0329 32910776

[B39] Zhang Y. T. Liang R. Wang Z. P.. (2005). Comparison of Different Color Models in Digital Image of Tongue Color of 884 Patients Undergoing Physical Examination. Chin. J. Basic Med. Traditional Chin. Med. 11 (3), 3.

[B40] Zhang D. Zhang H. Zhang B. (2017). “Tongue Segmentation by Fusing Region-Based and Edge-Based Approaches,” in Tongue Image Analysis. Eds. Zhang D. Zhang H. Zhang B. (Singapore: Springer), 115–131. doi: 10.1007/978-981-10-2167-1_7

[B41] Zhu C. Tabas I. Schwabe R. F. Pajvani U. B. (2021). Maladaptive Regeneration - the Reawakening of Developmental Pathways in NASH and Fibrosis. Nat. Rev. Gastroenterol. Hepatol. 18, 131–142. doi: 10.1038/s41575-020-00365-6 33051603 PMC7854502

